# Electrohydrodynamic-Jet (EHD)-Printed Diketopyrrolopyroole-Based Copolymer for OFETs and Circuit Applications

**DOI:** 10.3390/polym11111759

**Published:** 2019-10-26

**Authors:** Kyunghun Kim, Se Hyun Kim, Hyungjin Cheon, Xiaowu Tang, Jeong Hyun Oh, Heesauk Jhon, Jongwook Jeon, Yun-Hi Kim, Tae Kyu An

**Affiliations:** 1 Department of Chemical Engineering, Pohang University of Science and Technology, Pohang 37673, Korea; kim3034@purdue.edu; 2 School of Chemical Engineering, Yeungnam University, 280 Daehak-Ro, Gyeongsan, Gyeongbuk 38541, Korea; shkim97@yu.ac.kr (S.H.K.); tangxiaowu@naver.com (X.T.); 3 Department of Chemistry and RIGET, Gyeongsang National University, Jin-ju 660-701, Korea; tott22@hanmail.net; 4 Department of Polymer Science & Engineering, Korea National University of Transportation, 50 Daehak-Ro, Chungju 27469, Korea; ohjh1206@naver.com; 5 Department of Electrical, Information and Communication Engineering, Mokpo National University, Mokpo 530729, Korea; kindro1@mokpo.ac.kr; 6 Department of Electrical and Electronics Engineering, Konkuk University, Seoul 05029, Korea

**Keywords:** electrohydrodynamic-jet printing, diketopyrrolopyrrole, organic field-effect transistors, compact model, ring oscillator

## Abstract

We report the employment of an electrohydrodynamic-jet (EHD)-printed diketopyrrolopyrrole-based copolymer (P-29-DPPDTSE) as the active layer of fabricated organic field-effect transistors (OFETs) and circuits. The device produced at optimal conditions showed a field-effect mobility value of 0.45 cm^2^/(Vs). The morphologies of the printed P-29-DPPDTSE samples were determined by performing optical microscopy, X-ray diffraction, and atomic force microscopy experiments. In addition, numerical circuit simulations of the optimal printed P-29-DPPDTSE OFETs were done in order to observe how well they would perform in a high-voltage logic circuit application. The optimal printed P-29-DPPDTSE OFET showed a 0.5 kHz inverter frequency and 1.2 kHz ring oscillator frequency at a 40 V supply condition, indicating the feasibility of its use in a logic circuit application at high voltage.

## 1. Introduction

A variety of developed polymeric semiconductors have shown good electrical performances and, in many cases, outperform their metal oxide (ZnO, etc.) and amorphous silicon counterparts [1,2,3]. The planar structures of the polymeric chains have been shown to give rise to increased extents of molecular self-assembly and well-developed microcrystalline domains, and thereby high electrical performances with field-effect mobilities (*μ*_FET_s) of over 70 cm^2^/(V∙s) in organic field-effect transistors (OFETs) [4,5]. Various printing technologies, such as capillary force lithography and roll-to-roll coating, as well as aerosol jet, inkjet, and electrohydrodynamic-jet (EHD) printing, have been recently used to deposit and pattern polymeric semiconductors [6,7,8,9].

EHD printing has proven to be a particularly cost-effective and simple printing tool [10]. This printing tool generates a strong electrostatic field between a nozzle tip and a substrate to eject ink directly from the nozzle, and, as a result, can produce diverse fine patterns of polymeric semiconductors from the micro- to nano-scale without the need for any pre-patterned topographical or chemical pattern [11,12,13]. In addition, EHD printing is suitable for printing semiconducting polymers because of the relatively high viscosities of such polymer inks, compared to those of small-molecule inks; as a result, this printing tool can produce high-resolution patterns of polymeric semiconductors [14].

**Figure 1:**
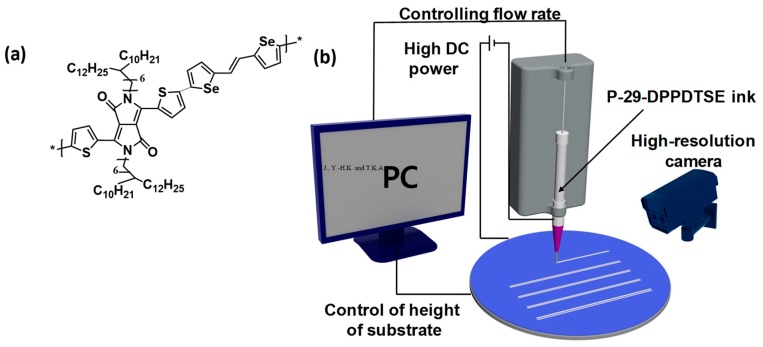
(**a**) Molecular structure of P-29-DPPDTSE and (**b**) schematic diagram showing the electrodynamic (EHD) printing process.

In this study, we demonstrated EHD printing of a diketopyrrolopyrrole (DPP)-based copolymer, namely poly[2,5-bis(7-decylnonadecyl)pyrrolo[3,4-c]pyrrole-1,4(2*H*,5*H*)-dione-(E)-(1,2-bis(5-(thiophen-2-yl)selenophen-2-yl)ethene) (P-29-DPPDTSE), as shown in Figure 1a, to fabricate patterned OFETs. DPP-based copolymers constitute a kind of donor-acceptor (D-A) conjugated copolymer and have been studied intensively due to their planar backbones and strong inter/intramolecular charge transport [15,16]. Various DPP-based copolymers have been investigated for use in OFETs [5,16,17,18,19,20,21]. Of these copolymers, for this study, we chose P-29-DPPDTSE in a previously reported investigation due to its high field-effect mobility (*μ*_FET_) [5]. The EHD-printed P-29-DPPDTSE films made using this copolymer showed moderate surface roughness and homogeneous crystalline morphology. EHD-printed films showed typical p-type behavior with *μ*_FET_s of about 0.45 cm^2^/(V∙s) when annealed at 200 °C. To investigate the properties of printed P-29-DPPDTSE OFETs, specifically to study the morphological properties of their films, we analyzed them using optical microscopy (OM), atomic force microscopy (AFM), and X-ray diffraction (XRD). In addition, to confirm the feasibility of using EHD-printed P-29-DPPDTSE OFETs in a high-voltage logic circuit operation, a compact model library to describe the electrical behaviors of P-29-DPPDTSE OFETs was developed. By implementing the developed compact model library into a circuit simulator such as SPICE, we successfully evaluated the high-voltage-logic operation of a p-type organic inverter and ring oscillator with, respectively, 1.2 kHz operating frequencies in a 40 V supply condition.

## 2. Experimental

### 2.1. Materials

P-29-DPPDTSE was prepared by using the previously reported method [5]. The number average molecular weight and polydispersity index (PDI) of the polymer were measured by carrying out room-temperature gel permeation chromatography with a polystyrene standard calibration together with tetrahydrofuran as the eluent. The number average molecular weight and PDI of P-29-DPPDTSE were determined to be 35,700 g/mol and 1.65, respectively.

### 2.2. Morphological Characterization

The crystal structures of EHD-printed P-29-DPPDTSE were characterized using two-dimensional grazing-incidence wide-angle X-ray scattering (2D-GIWAXS) performed at the 3C beamline of the Pohang Accelerator Laboratory (PAL), Pohang, Korea. AFM experiments were conducted using a Multimode Illa (Veeco Inc. PL, USA) operating in tapping mode with a silicon cantilever. The P-29-DPPDTSE samples used in the XRD and AFM studies were printed using the EHD jet on an octadecyltrichlorosilane (ODTS)-modified silicon wafer to mimic the device fabrication process and then dried under a vacuum at room temperature. After this deposition of the polymer, the samples were annealed at 200 °C for 10 min to test the effect of thermal annealing.

### 2.3. Device Fabrication and Measurements

To fabricate OFETs based on P-29-DPPDTSE, we used heavily N-doped silicon with a 100 nm-thick thermally grown layer of SiO_2_ as a dielectric. The capacitance of the dielectric layer was 30 nFcm^−2^. OFET properties of the P-29-DPPDTSE were characterized in a bottom gate/top contact architecture with gold source/drain electrodes. The surface of the silicon oxide, before being modified with ODTS, was first cleaned with a piranha solution [H_2_O_2_ (40 mL)/concentrated H_2_SO_4_ (60 mL)] for 20 min at 280 °C, rinsed with distilled water several times, and treated with ozone for 15 min. Then the SiO_2_ dielectric was treated with an ODTS monolayer and with toluene for 90 min at room temperature. EHD printing was then used to deposit the P-29-DPPDTSE semiconductor layer on the ODTS-treated SiO_2_ dielectric. For the fabrication of printed OFETs, EHD-jet printing of P-29-DPPDTSE was conducted using an EHD printer (Enjet, Suwon, Korea) operated using its cone-jet mode. A metallic nozzle holder attached to a glass syringe in the EHD printer was filled with a 1 wt% P-29-DPPDTSE solution in chloroform. The P-29-DPPDTSE solution was ejected at a flow rate of 0.10 μL/min using a syringe pump through a nozzle with a diameter of 50 μm. To apply an electrostatic field between the nozzle and the Au substrate ground, a supply voltage of 2.5 kV was generated by an installed power supply. The printing speed and working distance were fixed at 5 mm/s and 100 μm, respectively. The entire process was interfaced with a computer and monitored using a CCD (charge-coupled device) camera. Finally, Au source and drain electrodes (100 nm thickness) were deposited by carrying out thermal evaporation through a shadow mask (with the channel region having a length (L) of 50 μm, and width (W) of 1000 μm). The OFET devices were annealed at 200 °C for 10 minutes under a nitrogen atmosphere. OFET device measurements were taken in an N_2_-purged glove box (H_2_O, O_2_ < 0.1 ppm) using both Keithley 2400 and 236 source/measure units. The *μ*_FET_ values were extracted in the saturation regime from the slope of the source-drain current.

### 2.4. Computational Simulations

In the development process of inorganic semiconductors such as silicon, the industry uses the evaluation results of dynamic circuit characteristics in actual application circuits as the main indexes. Therefore, in OFET development, it is important to examine the performance when applied to more practical application circuits by performing dynamic AC (alternating current) analysis in addition to device measurement and analysis in steady-state DC (direct current) state. In this process, an inverter circuit, a standard cell, which is the most basic logic application, is mainly used for benchmarking between different technologies. Other standard cell circuits, such as NAND and NOR, could be expanded, but in this work, an inverter was selected as the main benchmark circuit as in many inorganic semiconductor technology development works.

In order to apply the P-29-DPPDTSE-based OFET to the integrated circuits, it was essential to provide a design environment using an electronic design automation (EDA) tool, and to do so, a compact model capable of describing electrical characteristics such as I-V (current-voltage) and C-V (capacitance and voltage) under various design bias conditions of OFETs was required. A compact model consisting of analytical equations was implemented in the SPICE (Simulation Program with Integrated Circuit Emphasis) design tool. Then, the circuit optimization was performed by simulating the characteristics of the design circuit for various bias conditions and device sizes in conjunction with other circuit-constituting elements. In order to verify the high-voltage logic circuit characteristics of the synthesized OFETs, Synopsys’ HSPICE was used, specifically to simulate dynamic circuit characteristics in conjunction with extracted BSIM4 (Berkeley Short-channel IGFET (Insulated-Gate Field-Effect Transistor) Model 4) model parameter libraries for describing electrical characteristics of each device. Note that BSIM4, which is a widely used industry-standard model and provides various fitting parameters, was used. For the dynamic AC analysis, it is important to extract the model parameters for the electric behaviors. The feasibility of applying BSIM4 to OFETs has already been confirmed in our previous work [22]. Note that, in the developed model library, the gate leakage current and the parasitic capacitance between the gate and source/drain can be negligible due to the use of a very thick gate oxide and due to the fringe field due to the use of a very large device area, respectively.

## 3. Results and Discussion

**Figure 2:**
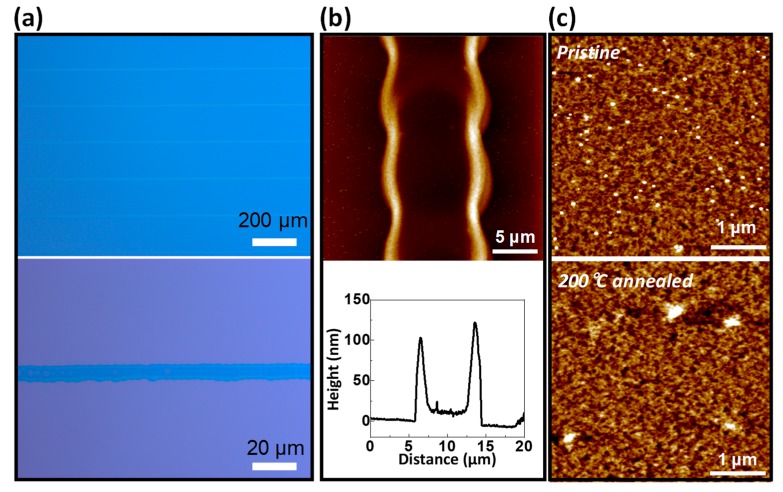
(**a**) Optical microscopy (OM) images and (**b** and **c**) height-mode atomic force microscopy (AFM) images of EHD-printed P-29-DPPDTSE patterns.

**Figure 3:**
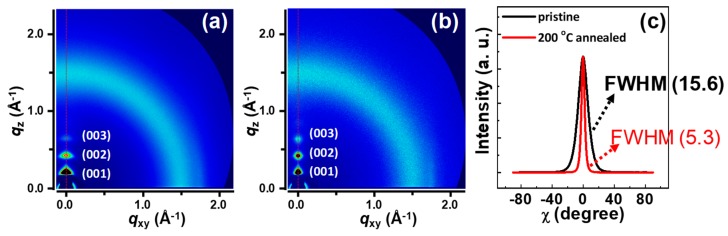
(**a**,**b**) XRD of (a) pristine and (**b**) thermally annealed EHD-printed P-29-DPPDTSE patterns, and (**c**) pole figures extracted from the (001) peaks of these XRD results. The peak intensity in each case was normalized by the crystal volume.

EHD printing was used to fashion P-29-DPPDTSE as the active layers of the OFETs (Figure 1b and Figure 2a). Previous studies by the Kim group optimized the thermal annealing conditions for the device fabrication [5]. The morphologies of the EHD-printed P-29-DPPDTSE lines were intimately related to its electrical properties when used as active layers in the OFETs. Patterned P-29-DPPDTSE lines were characterized by using AFM (Figure 2b,c). The pristine P-29-DPPDTSE sample and that annealed at 200 °C showed root-mean-square (RMS) roughness values of 2.20 nm and 2.03 nm, respectively, with the annealed sample more clearly showing a granular morphology. Molecular packing of P-29-DPPDTSE in the printed lines was investigated by taking XRD measurements of them (Figure 3). Both the pristine and annealed P-29-DPPDTSE lines showed (001), (002), and (003) XRD peaks along the out-of-plane direction. The pristine P-29-DPPDTSE sample yielded a (001) diffraction peak at q = 0.23 Å^−1^, with an interlayer distance of 27.3 Å. By contrast, the annealed P-29-DPPDTSE sample yielded a (001) peak at q = 0.22 Å^−1^, with an increased interlayer distance of 28.6 Å. This result may have been due to a straightening of bent side chains as a result of thermal annealing.

To obtain more accurate information about the crystallinity of the EHD-printed P-29-DPPDTSE patterns, coherence lengths were extracted from the (001) diffraction peaks in the out-of-plane profiles of the samples (Figure 3c), with these lengths determined as 2π/FWHM (FWHM: full width at half-maximum of the peak). As summarized in Table 1, compared to the (001) peak for the pristine P-29-DPPDTSE sample, that for the annealed sample showed a lower FWHM value, yielding a greater coherence length of 400.7 Å. This result was indicative of a smaller grain boundary of the annealed P-29-DPPDTSE sample, which could reduce the barrier for changes to efficient transport [23,24].

**Figure 4:**
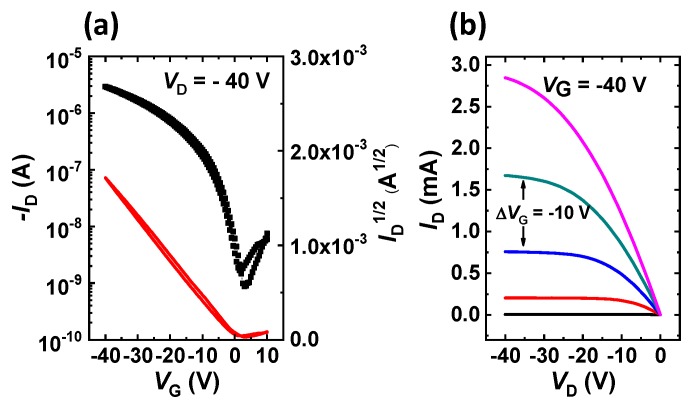
(**a**) Transfer and (**b**) output curves from an annealed P-29-DPPDTSE organic field-effect transistor (OFET).

**Figure 5:**
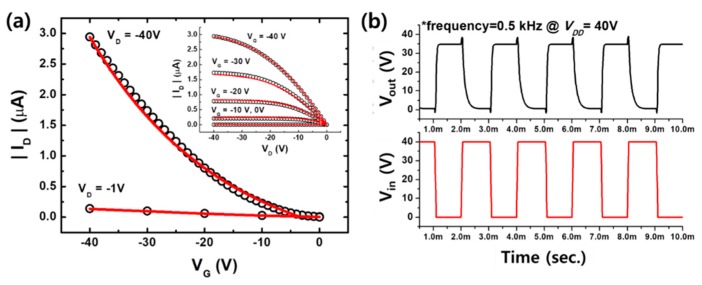
(**a**) The results of fitting BSIM4 model parameters to OFET device data. Symbols and lines represent the measurements and Simulation Program with Integrated Circuit Emphasis (SPICE) simulation results, respectively. (**b**) Transient waveform of a P-29-DPPDTSE OTFT-based inverter.

The electrical characteristics of the EHD-printed P-29-DPPDTSE films (Figure 4) were evaluated by fabricating typical bottom-gate/top-contact OFETs. The P-29-DPPDTSE OFET devices annealed at 200 °C showed (Table 2) a hole *μ*_FET_ of 0.45 cm^2^/(V∙s) with an on/off ratio of 3.0 × 10^3^, while the OFETs without thermal annealing showed a much lower hole *μ*_FET_ of 0.09 cm^2^/(V∙s) with an on/off ratio of 6.3 × 10^2^. To better understand the circuit operation, the electrical behaviors of the synthesized OFETs were modeled by using the Berkeley Short-Channel IGFET Model 4 (BSIM4) [22,25]. As shown in Figure 5a, the extracted BSIM4 model (lines) reproduced the measured P-29-DPPDTSE OFET (symbols) quite well and could accurately simulate the circuits in conjunction with a circuit simulator, such as SPICE. As shown in Figure 5b, the logic-circuit performances of the p-type OFET inverter with an operating frequency of 0.5 KHz were evaluated; these values were well modeled by the developed BSIM4 model library.

**Figure 6:**
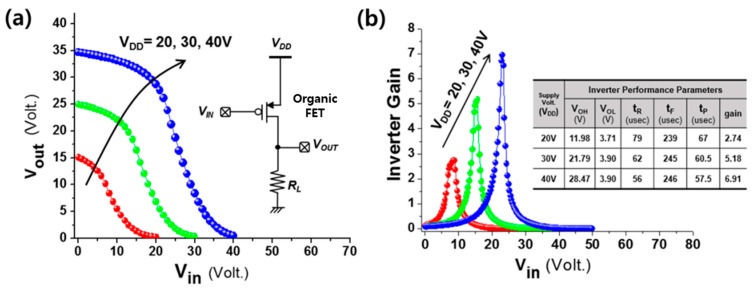
Inverter design having a driver transistor together with a resistive load. (**a**) Voltage-transfer characteristics. (**b**) Inverter gains for various supply voltages ranging from 20 V to 40 V. Also included is a table summarizing inverter performance measures for the different supply voltage conditions.

The inverter gate we tested basically consisted of one resistive load, R_L_ (∼60 MΩ), and one driver OFET with W/L values of 1000/50 μm (see inset schematic of Figure 6a). When the input voltage (*V_in_*) was in a low-voltage state (0 V), the p-type OFET acted as a shortened switch and pulled the output voltage (*V_out_*) to the DC supply, supply voltage (*V_DD_*). On the other hand, the transistor was in the open state, and finally, *V_out_* was pulled down by the resistor *R_L_* to ground as *V_in_* reached a high-voltage state (*V_DD_*). Figure 6a,b show, respectively, the acquired voltage-transfer curves and the inverter gains of the designed inverter for supply voltages ranging from 20 V to 40 V. Performance parameters such as the minimum output high voltage (*V_OH_*), maximum output low voltage (*V_OL_*), rising time (*t_R_*), falling time (*t_F_*), and propagation delay time (*t_P_*) of the inverter for various supply voltages are also summarized in the table of Figure 6b [26].

When the circuit performance of the EHD-printed P-29-DPPDTSE OFET in this work is compared to the circuit performance of the Poly(quinacridone-quinoxaline), PQCQx, polymer-based OFET from our previous work [25], it is observed that EHD-printed P-29-DPPDTSE OFET shows much better performance than the previous work. Since the two OFET devices target different operating voltages, it is difficult to make a direct comparison. Therefore, we compared the two OFET devices at the same over-drive voltage defined by the threshold voltage and the supply voltage difference for a fair comparison. Comparing the logic inverter gain to a similar over-drive voltage case (*V_DD_* = 40 V in this work), the gain of the EHD-printed P-29-DPPDTSE OFET is 6.91, and the gain of the PQCQx OFET is 3.20, which shows that the EHD-printed P-29-DPPDTSE OFET has better logic circuit performance.

**Figure 7:**
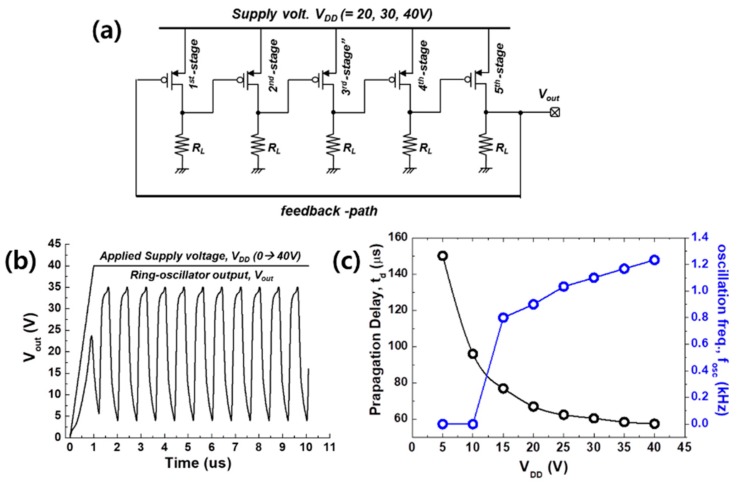
(**a**) Schematic diagram of a 5-stage ring oscillator circuit using a resistive load inverter, in which L/W = 50/1000 μm, and *R_L(load)_* ~60 MΩ. (**b**) The transient (dynamic) simulation results for the experiment in which *V_DD_* was increased with time; here, the organic BSIM4 model was used. (**c**) Propagation delay and ring oscillator *f_OSC_* as a function of *V_DD_*.

Additionally, as depicted in Figure 7, a five-stage ring oscillator composed of a designed inverter with resistive load was also evaluated. The output terminal (*V_out_*) of the last stage was connected to the input terminal of the first stage with a feedback path. An output oscillation frequency (*f_OSC_*) of 1.2 kHz was measured for a *V_DD_* of 40 V, and *f_OSC_* increased as the supply voltage (*V_DD_*) was increased, as plotted in Figure 7c.

## 4. Conclusions

In this work, a conjugated P-29-DPPDTSE polymer based on both a DPP unit and a selenophene vinylene selenophene unit was applied in EHD-printed OFETs and simulated in circuits. The thermally annealed OFET device showed a high field-effect mobility value at 0.45 cm^2^/(Vs). The morphologies of the printed P-29-DPPDTSE samples were determined by carrying out OM, XRD, and AFM experiments, and a designed inverter and ring oscillator using P-29-DPPDTSE-based OFETs were shown to successfully realize a logic operation with, respectively, frequencies of 0.5 kHz and 1.2 kHz at a 40 V supply condition.

## Figures and Tables

**Table 1:** Results of the crystallographic analysis of E-jet-printed P-29-DPPDTSE crystals. *d*(001) denotes the *d*-spacing value of the (001) plane. The coherence lengths were determined from the full width at half-maximum of the peak (FWHM) values of the (001) peaks in Figure 3.

Conditions	Crystallographic Parameters	Value
pristine	*q* (Å^−1^) at (001) plane	0.23
	*d*-spacing (Å)	27.32
	FWHM (Å^−1^)	0.03005
	Coherence length (Å)	209.0
200 °C annealed	*q* (Å^−1^)	0.22
	*d*-spacing (Å)	28.56
	FWHM (Å^−1^)	0.01568
	Coherence length (Å)	400.7

**Table 2:** Performance measures of various OFETs with different PQCTQx films.

P-29-DPPDTSE	Mobility (cm^2^/V∙s)	On/off
As-cast	0.09	6.3 × 10^2^
Annealed at 200 °C	0.45	3.0 × 10^3^
